# Artificial intelligence for distinguishment of hammering sound in total hip arthroplasty

**DOI:** 10.1038/s41598-022-14006-2

**Published:** 2022-06-14

**Authors:** Yasuhiro Homma, Shun Ito, Xu Zhuang, Tomonori Baba, Kazutoshi Fujibayashi, Kazuo Kaneko, Yu Nishiyama, Muneaki Ishijima

**Affiliations:** 1 Department of Orthopaedic Surgery, Juntendo University, 2-1-1 Hongo, Bunkyo-ku, Tokyo, 113-0033 Japan; 2 Faculty of Informatics and Engineering, The University of Electro-Communications, Tokyo, Japan; 3 Medical Technology Innovation Center, Juntendo University, Tokyo, Japan

**Keywords:** Bone, Orthopaedics

## Abstract

Recent studies have focused on hammering sound analysis during insertion of the cementless stem to decrease complications in total hip arthroplasty. However, the nature of the hammering sound is complex to analyse and varies widely owing to numerous possible variables. Therefore, we performed a preliminary feasibility study that aimed to clarify the accuracy of a prediction model using a machine learning algorithm to identify the final rasping hammering sound recorded during surgery. The hammering sound data of 29 primary THA without complication were assessed. The following definitions were adopted. Undersized rasping: all undersized stem rasping before the rasping of the final stem size, Final size rasping: rasping of the final stem size, Positive example: hammering sound during final size rasping, Negative example A: hammering sound during minimum size stem rasping, Negative example B: hammering sound during all undersized rasping. Three datasets for binary classification were set. Finally, binary classification was analysed in six models for the three datasets. The median values of the ROC-AUC in models A–F among each dataset were dataset a: 0.79, 0.76, 0.83, 0.90, 0.91, and 0.90, dataset B: 0.61, 0.53, 0.67, 0.69, 0.71, and 0.72, dataset C: 0.60, 0.48, 0.57, 0.63, 0.67, and 0.63, respectively. Our study demonstrated that artificial intelligence using machine learning was able to distinguish the final rasping hammering sound from the previous hammering sound with a relatively high degree of accuracy. Future studies are warranted to establish a prediction model using hammering sound analysis with machine learning to prevent complications in THA.

## Introduction

Technologies based on machine learning algorithms, often called artificial intelligence (AI), are beginning to be used in everyday life in various ways. Machine learning methods, especially supervised learning, are used to solve classification issues in the healthcare field. Several recent studies have shown that machine learning and big data mining approaches effectively improve screening, prediction, selection, and diagnosis in healthcare^1–6^.

Very recently, AI has also been assessed in the orthopaedic field, especially in image diagnosis^6,7^. One study demonstrated that AI performed at a human level in interpretating 256,000 orthopaedic radiographs^6^. Another study showed that AI exhibits a diagnostic ability similar to that of orthopaedists and a performance superior to that of radiologists in distinguishing anteroposterior wrist radiographs with distal radius fractures from normal images under limited conditions^7^.

Although few studies have assessed whether AI could contribute to total hip arthroplasty (THA)^8–11^, it is expected that AI could help surgeons and improve the clinical outcome of THA. One of the major unresolved complications in THA is intra-operative fracture^12,13^, which reportedly occurs at a rate of 1.5–27.8%^12^. In addition to recent developments such as three-dimensional (3D) templating of the soft tissue and modern implant shapes, studies have focused on sound analysis during insertion of the cementless stem to decrease the incidence of intra-operative femoral fracture^14,15^. One study identified the specific pattern (in a non-quantitative manner) of the hammering sound during cementless stem insertion related to stem subsidence and intra-operative fracture^15^. However, the nature of the hammering sound of cementless stem insertion is complex and varies widely owing to numerous possible variables such as the bone quality, femoral morphology, implant design, and hammering method. Thus, it is difficult to analyse the hammering sound using ordinary statistics and to clinically apply such sound analysis to prevent complications such as intra-operative fracture in THA.

Therefore, we hypothesized that AI could contribute to the understanding of and analyse the hammering sound of cementless stem insertion. As no study has used AI to analyse the sound during THA, we performed a preliminary feasibility study that aimed to clarify the accuracy of a prediction model using a machine learning algorithm to identify the final rasping hammering sound recorded during surgery.

## Material and methods

### Patients

All procedures performed in this study involving human participants were in accordance with the ethical standards of the institutional and/or national research committee and the 1964 Helsinki declaration and its later amendments or comparable ethical standards. The study protocol was approved by the Ethics Committee of Juntendo University. Informed consent was obtained in a manner approved by the Ethics Committee from all individual participants included in this study. The sound data recorded during 36 primary THA procedures performed on patients who agreed to participate in this study were initially included (sampling frequency: 44.1 kHz). The exclusion criteria were stem subsidence (> 2 mm) within 3 weeks post-operatively (n = 7) and intra- or post-operative femoral fracture (n = 0). The sound data from 23 women and six men (age range 48–89 years) were finally included.

### Surgical procedure

The operations were performed by one member of the hip specialist team via the direct anterior approach with different implants, including a cementless proximally hydroxyapatite (HA)-coated stem (Accolade 2; Stryker, Tokyo, Japan), Taperloc Complete Microplasty (Zimmer Biomet, Warsaw, IN, USA), full-HA porous triple tapered stem (Twinsys; Matys Ltd., Bettach, Switzerland), and meta-diaphyseal anchoring short-stem system (Optimys; Mathys Ltd., Bettlach, Switzerland). The direct anterior approach with the patient in the supine position on a surgical traction table was performed. Intra-operative radiography was used to confirm the alignment and size of the implant. All patients were allowed full weight-bearing initiated on the first day post-operatively with standardized protocol.

### Sound data collection during THA

**Figure 1:**
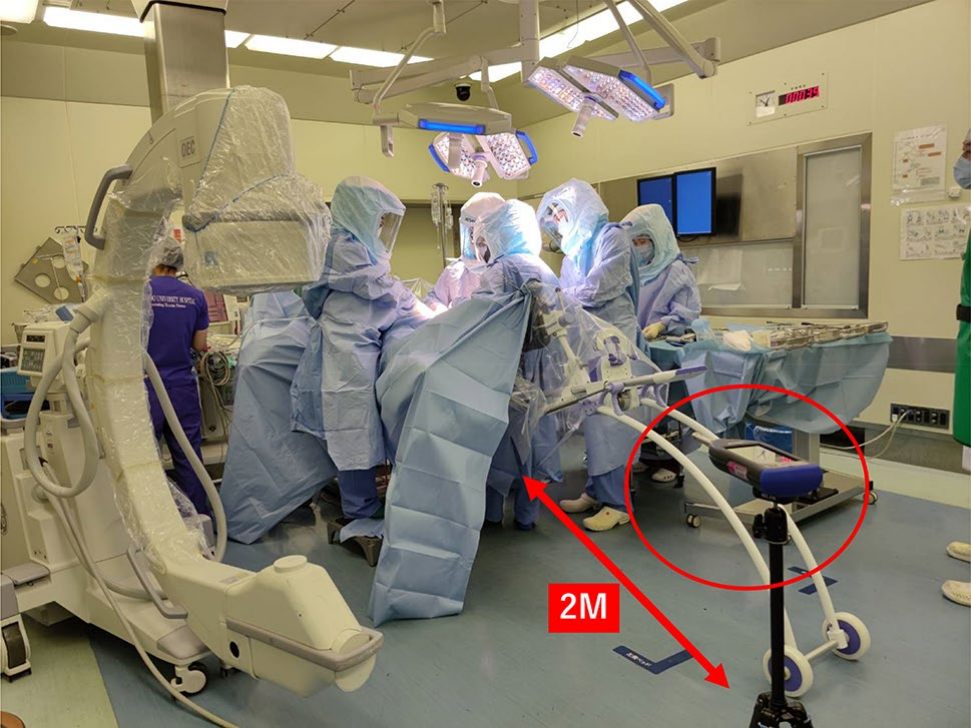
A highly sensitive sound level meter (LA-7500; Onosokki, Kanagawa, Japan) was used to record the hammering sound of stem insertion. In all cases, the sound level meter was set on a tripod mount at 1 meter high and 2 meters away from the surgical table in the same operation room.

A highly sensitive sound level meter (LA-7500; Onosokki, Kanagawa, Japan) was employed to record the hammering sound of stem insertion. In every case, the sound level meter was set on a tripod mount at 1 m high and 2 m away from the surgical table in the same operation theatre (Fig. 1). The rasping procedure was performed by standard technique. The size of rasping was started from the smallest one and sized up one by one. Although the exact protocol of hammering technique was not set, the standard hammering technique was performed. Range of 40–110 dB using Z frequency weighting (flat-weighted filter) and fast time weighting at a sampling rate of 64 kHz and 16-bit sampling depth were set for recording.

### Signal extraction of the hammering sound

As the sound data consisted of various sounds such as the hammering sound, conversation, monitoring sounds, and background noise, the following method was applied to extract the signals of the hammering sound made by the stem rasping.

**Figure 2:**
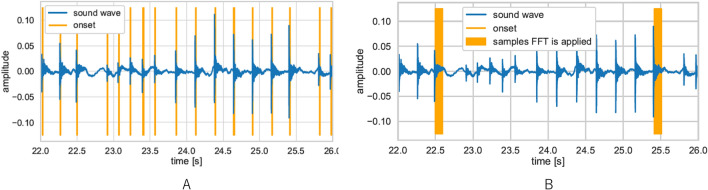
Signal extraction of the hammering sound. Step 1; Automatic detection of the signals of the hammering sound using the python library of the voice processing system (Librosa) (2A). Step 2; Every automatically detected sound was reviewed by a human, and all sounds other than the hammering sound were deleted manually (2B).

**Figure 3:**
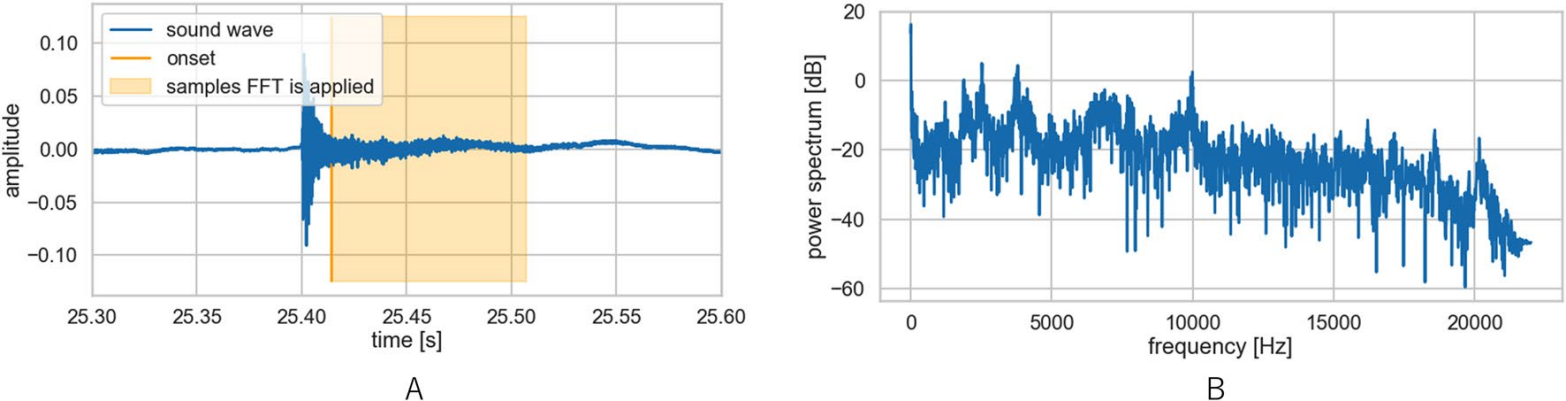
Signal extraction of the hammering sound. Step. 3; The hammering sound was assessed during the period from the onset to 0.093 s (3A). Input variable setting; the sound data was analysed by Fast Fourier transform analysis (3B).

- *Step* 1: Automatic detection of the signals of the hammering sound using the python library of the voice processing system (Librosa) (Fig. 2A).
- *Step* 2: Every automatically detected sound was reviewed by a human, and all sounds other than the hammering sound were deleted manually (Fig. 2B).
- *Step* 3: The hammering sound was assessed during the period from the onset to 0.093 s (Fig. 3A).
- *Step* 4: The final hammering sound of the stem rasping for each stem size was categorized. When the final hammering sound was overlapped with conversation or other noise, those sounds were excluded.

### Datasets for machine learning

To prepare the dataset for machine learning, the following definitions were adopted.

- Undersized rasping: all undersized stem rasping before the rasping of the final stem size.
- Final size rasping: rasping of the final stem size.
- Positive example: hammering sound during final size rasping.
- Negative example A: hammering sound during minimum size stem rasping.
- Negative example B: hammering sound during all undersized rasping.

For example, in an operation ID, Optimys stem was used, undersized rasping was tried with size index [0, 2, 3, 4] in ascending order, and final size rasping was determined as 4 by a skilled hip specialist, which resulted in no subsidence. In this case, positive examples (n = 9) were extracted from the hammering sound data during stem size 4. Negative examples A (n = 8) were extracted from the hammering sound data during stem size 0. Negative examples B (n = 19) were extracted from the hammering sound data during stem sizes [0, 2, 3]. To distinguish hammering sound data between positive example and negative example B would be more difficult than to distinguish hammering sound between positive example and negative example A, because sizes of 3 and 4 are closer than sizes of 0 and 4.

In this study, a total of 523 hammering sounds were analysed and the following three datasets for binary classification were set.

- Dataset A: no subsidence and cases with the Accolade 2 stem (positive example: n = 109, negative example A: n = 50).
- Dataset B: no subsidence and cases with the Accolade 2 stem (positive example: n = 109, negative example B: n = 207).
- Dataset D: no subsidence and cases with all stem types (positive example: n = 168, negative example B: n = 355). Dataset D includes various hammering sound data of various stem types. Therefore, to distinguish hammering sound data between positive example and negative example B would be most difficult in the three datasets A, B, and D.

### Evaluation settings

A test operation ID to evaluate the prediction accuracy of trained models was randomly selected over all the operation IDs, and the hammering sound data within the selected operation ID were used as test data, while the others were set as training data (leave-one-out cross-validation). The classification accuracy was measured using the area under the receiver operating characteristic curve (ROC-AUC).

### Input variable settings

The sound data of the ith hammering sound were defined as $A_{i} \in {\mathbb{R}}^{4096}$ (4096 sampling points). The sound data after Fast Fourier transform (FFT) analysis (Fig. 3B) was defined as $P_{i} \in {\mathbb{R}}^{4096}$. The power spectral *Pi*(ω) (ω ∈ [0; 22000], sampling points) of all or partial frequency bands under the Nyquist frequency *λ*_S_/2 = 22.05 (kHz) were used as input variables.

### Discriminative model settings

Binary classification was analysed in the following six models for the three datasets (A, B, and D). The hyperparameter for L2 regularization was *C* = 0.1.

- Model A: logistic regression (LR). LR analysis was performed with the input variable ${P}_{i}\in {\mathbb{R}}^{4096} \mathrm{and}$ output variable ${\mathscr{Y}}_{i}\in \left\{0, 1\right\}.$
- Model B: truncated singular value decomposition (tSVD) + LR. Dimension reduction was performed from ${P}_{i}\in {\mathbb{R}}^{4096}$ to ${\mathcalligra{p}}_{i}\in {\mathbb{R}}^{10}$ by tSVD. LR analysis was performed with the input variable ${\mathcalligra{p}}_{i}\in {\mathbb{R}}^{10}$ and output variable ${\mathscr{Y}}_{i}\in \left\{0, 1\right\}.$
- Model C: ensemble learning (LRs). All the hammering sound learning data were randomly divided into (*K* − 1) subdata, where *K* − 1 is the number of operation IDs used for training data, and (*K* − 1) weak learners (LRs) were trained using subdata. The prediction probability of (y = 1) in the *i*th test hammering sound of the *k*th weak learner was defined as ${p}_{i}^{(k)}$. The final prediction probability of the ith test hammering sound was determined by voting of $\frac{1}{K-1}{\sum }_{k=1}^{K-1}{p}_{i}^{(k)}$ in the (K − 1) weak learners.
- Model D: ensemble learning (tSVD + LRs). The difference of model D from model C is that input variables for training (K − 1) weak learners were changed from ${P}_{i}\in {\mathbb{R}}^{4096}$ to ${\mathcalligra{p}}_{i}\in {\mathbb{R}}^{10}$ by a dimension reduction method (tSVD), similar to the setting of model B. Other settings were the same as for model C.
- Model E: ensemble learning (LRs). Hammering sound data may vary depending on various factors including patients’ backgrounds, stem types, skilled operators, and sound-collected operating rooms. Considering this, training data were not merged with different operations, but a weak learner was trained operation-wise. Other settings were the same for model C.
- Model F: ensemble learning (tSVD + LRs). The difference of model F from model E is that input variables for training (K − 1) weak learners were changed from ${P}_{i}\in {\mathbb{R}}^{4096}$ to ${\mathcalligra{p}}_{i}\in {\mathbb{R}}^{10}$ by a dimension reduction method (tSVD), similar to the setting of model B. Other settings were the same as for model C.

### Ethical approval

All procedures performed in this study involving human participants were in accordance with the ethical standards of the institutional and/or national research committee and the 1964 Helsinki declaration and its later amendments or comparable ethical standards. The study protocol was approved by the Ethics Committee of our institution.

### Consent to participate

Informed consent was obtained in a manner approved by the Ethics Committee from all individual participants included in this study.

## Results

**Figure 4:**
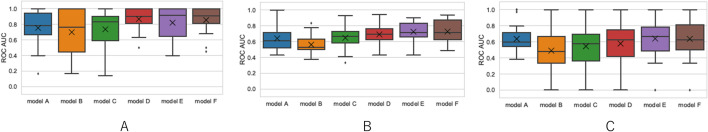
Results of ROC-AUC in models A–F among each dataset.

For dataset A, models D and F performed better than the other models; the median values of the ROC-AUC in models A–F were 0.79, 0.76, 0.83, 0.90, 0.91, and 0.90, respectively (Fig. 4A). For dataset B, models E and F performed better than the other models; the median values of the ROC-AUC in models A–F were 0.61, 0.53, 0.67, 0.69, 0.71, and 0.72, respectively (Fig. 4B). For dataset C, models E and F performed better than the other models; the median values of the ROC-AUC in models A–F were 0.60, 0.48, 0.57, 0.63, 0.67, and 0.63, respectively (Fig. 4C).

## Discussion

Recent technology has used FFT analysis of the hammering sound during cementless stem implantation in THA as a novel method to decrease the incidence of complications^15^. However, the complex nature of the hammering sound of cementless stem insertion is a large obstacle to the development of this technology. We conducted this preliminary feasibility study to assess the accuracy of a prediction model using a machine learning algorithm to identify the final rasping hammering sound using only the hammering sound recorded during THA. Our study demonstrated that AI using machine learning distinguished the final rasping hammering sound from the former hammering sound with a relatively high degree of accuracy.

Although further development of hammering sound analysis is needed, the present results suggest that this may be a novel method to distinguish between correct and incorrect hammering sounds during the surgery to decrease complications in THA. So far, there are no reliable methods with which to predict THA complications such as intra-operative fracture and subsidence. To decrease the complications in THA, pre-operative 3D templating and intra-operative navigation have been used to avoid inadequate stem selection and achieve better stem positioning^16^. The accuracy of predicting the right stem size reportedly improves from 45.7 to 58.6% using 3D templating compared with 2D surgical planning^16^. However, although greater accuracy in stem size selection has been achieved, surgical errors such as excessive hammering may lead to intra-operative fracture, while inadequate hammering leads to subsidence post-operatively. Therefore, monitoring of appropriate hammering could represent an advanced method with which to prevent THA complications. A previous study analysed the acoustic frequency patterns and found that a natural hammering frequency of approximately 7 kHz is the most prominent frequency in patients without complications^15^. Furthermore, another study reported that a frequency of around 1 kHz better predicts an adequately sized stem using spectrographs^14^. However, these previous studies had limitations, as the results differed and could not be easily distinguished. This discrepancy is probably due to the complex nature of the hammering sound of cementless stem insertion. Moreover, the data in these previous studies were not objectively evaluated. Our results are unique because the data analysis was fully objective and systematically analysed by AI.

To achieve a higher degree of accuracy in detecting appropriate hammering sounds, it is necessary to perform machine learning with not only hammering sounds but also other information such as implant data. Our data showed a higher degree of accuracy in dataset B, which included only the one implant type, rather than dataset C, which included different cementless stems. The cementless stems used in the present study were the Accolade 2, Taperloc Complete Microplasty, Twinsys, and Optimys. Although the Accolade 2 and Taperloc are both classified as taper-wedge proximal coated stems, they have slight differences in the size, stem proportion, and contact point with the femoral cortex. The Twinsys is classified as a full-HA porous triple tapered stem, which is relatively longer than the taper-wedge proximal coated stem. The Optimys is classified as a short stem, which is completely different from the taper-wedge proximal coated stem. In addition to these morphological differences among stems, the characteristics of the hammering sounds may be influenced by the weight, contact point with the femoral cortex, and instruments used for each stem. Therefore, future studies are needed to investigate the effects of these variables on the sound pattern.

We believe that the hammering sound may also be influenced by patient background characteristics such as age, height, weight, bone quality, and femoral morphology. When the cementless stem is fixed in the canal of the femur, the stem-femoral complex is vibrated as one object. Therefore, there is no doubt that these variables affect the vibration of the stem-femoral complex, leading to the characterization of the hammering sound. The importance of various affects is also supported by our results that training multiple models in a stratified way to deal with various factors was better rather than training a single linear model using all the merged hammering sound data. The reasons why models E and F were better than other models are explained by follows. Models A and B attempted to predict positive or negative samples by training a single linear model, even though hammering sounds would be vary depending on operation IDs due to various factors such as patients’ backgrounds, stem types, skilled operators, and sound-collected operating rooms. Models C, D, E and F attempted to establish a nonlinear model via training multiple linear models (weak learners) and integrating their prediction results by voting. Especially, models E and F attempted to train weak learners operation-wise (including operator-wise and operating-room-wise) to take into account various factors of different operation IDs.

Furthermore, the FFT method also affected the results. In the present study, the analysed area was 0.093 s from the onset. The onset point and periods of the analysed area must affect the results of FFT. Therefore, future studies should also investigate the effect of the patient characteristics and the method of FFT analysis on the hammering sound. Since this study is conducted as a preliminary study to test AI for sound analysis, possible factors which might affect on the sound characteristics were not assessed in current study. Further investigation under control of those factors is needed.

This study had several limitations. First, relatively few operations were analysed. Although the maximum number of hammering sounds in a dataset was 523, hammering sound data from a larger number of operations might change the result. However, despite the small samples, the ROC-AUC value was higher than we expected. Second, it is unknown whether AI with machine learning can distinguish the hammering sound related to complications from the hammering sound in a case without complications. However, this study was designed to investigate the feasibility of using AI with machine learning to analyse the hammering sounds. Further establishment of a prediction model to prevent complications using hammering sounds in combination with other variables such as implant type and patient background characteristics must be studied in the future. Third, the positive example in this study was the hammering sound during final size rasping. We consider that there is likely to be variance among hammering sounds during final size rasping, which may have influenced our results. Correct definition of the positive example is mandatory for further investigations. Fourth, the area analysed for FFT was 0.093 from the onset. The analysis of a different area might lead to a different result. Future research is required to identify the most reliable area for FFT sound analysis.

## Conclusion

Our study demonstrated that AI using machine learning was able to distinguish the final rasping hammering sound from the previous hammering sound with a relatively high degree of accuracy. Future studies are warranted to establish a prediction model using hammering sound analysis with machine learning to prevent complications in THA.

## Data Availability

The datasets used and/or analysed during the current study available from the corresponding author on reasonable request.
